# Ultrasound diagnosis of age-related involutional changes in the lower third of face and neck to determine treatment techniques

**DOI:** 10.12669/pjms.37.1.3034

**Published:** 2021

**Authors:** Valentin Sharobaro, Sekina Alimova, Anna Telnova, Liudmila Shamanaeva

**Affiliations:** 1 Prof. Dr. Valentin Sharobaro, M.D. Plastic Surgeon, Department of Plastic & Reconstructive Surgery, Cosmetology and Cellular Technologies, N.I. Pirogov Russian National Research Medical University (RNRMU), Moscow, Russia, Federal Research Clinical Center of the FMBA, Moscow, Russia; 2 Dr. Sekina Alimova, M.D. Plastic Surgeon, Department of Plastic & Reconstructive Surgery, Cosmetology and Cellular Technologies, N.I. Pirogov Russian National Research Medical University (RNRMU), Moscow, Russia; 3 Dr. Anna Telnova, M.D. Ultrasound Specialist, Department of Ultrasound Diagnostics, Federal Research Clinical Center of the FMBA, Moscow, Russia; 4 Dr. Liudmila Shamanaeva, M.D. Plastic Surgeon, Assistant Professor, Dept. of Maxillofacial Surgery, I.M. Sechenov First Moscow State Medical University (Sechenov University), Moscow, Russia

**Keywords:** Ultrasound Diagnostics, Tissue Ptosis, Aged, Face, Neck, Mini-Invasive Treatment, Rejuvenation

## Abstract

**Objective:**

To develop a clinical technique for objective estimation of exact location and degree of participation of face and neck soft tissues in age-related deformations for determine effective mini-invasive treatment techniques.

**Methods:**

Ultrasound examination was performed in 2017-2019 at 63 patients with age-related face changes. Examination was done in the vertical position for determine the role, exact location and participation degree of different soft tissues of the face and neck in age-related changes.

**Results:**

Great diagnostic value of ultrasound examination of involutional changes in soft tissues of lower third of face and neck was noted to determine all causes of contour age-related deformities. These results were used to choose effective minimally invasive methods for correction.

**Conclusion:**

Ultrasound examination is a non-invasive, harmless, clinically available, inexpensive examination that allows to determine exact localization and degree of participation of different soft tissues of face and neck in age-related changes. That is very important for planning and use different minimally invasive techniques for facial and neck rejuvenation.

## INTRODUCTION

As the people’s life in the world has increased, they want to stay longer healthy, active, young and look good.^1^ Minimally invasive percutaneous correction techniques of age-related changes in soft tissues of face and neck, such as liposuction, platysmotomy, laser therapy and filaments have become more popular for the last a few decades.^2^-^4^ For their effectiveness and prevention of complications, objective visualization of the affected anatomical structures is necessary.

Historically, in plastic surgery, methods for objective assessment of deformities in the lower third of the face and neck are not so important for open surgical techniques, when a surgeon can correct his actions based on intraoperative situation. Good visualization of tissues is impossible during minimally invasive correction, but it is very important for objective evaluation of tissues causing the deformation and for correct planning the actions of plastic surgeon. The first report on facial muscle imaging using ultrasonography was published in 1988.^5^ However, due to technical limitations, the possibilities of ultrasound were not researched.

The aim of our research was to develop a clinical technique for objective estimation of exact location and degree of participation of face and neck soft tissues in age-related deformations for determine effective mini-invasive treatment tactics.

## METHODS

The prospective analysis of examination and treatment of 137 patients with aged-related involutional changes in soft tissues of lower third of face and neck was performed in Federal Research Clinical Center of the FMBA in the period from 2017 to 2019. The stages of the study were approved by the Ethics Committee of the N.I. Pirogov Russian National Research Medical University (protocol No. 170 dated by December 18, 2017). We have obtained the patent ‘Method for choosing treatment techniques for age-related involutive changes in the face and neck soft tissues’ RU 2710671 30.12.2019. Written consent of all patients to use the results of research and photos was obtained.

The age of patients was from 38 to 62 years. Among them, women were 121 (88.3%), men – 16 (11.7%). The average age was 49 ± 0.5 years (± standard deviation average). Presurgery ultrasound examination was done to 63 patients. The examination was performed using an ultrasound device Voluson E8Voluson E8 (Austria), a linear sensor 9 L, with a radiation frequency of 3-8 MHz. Ultrasound examination was done in the vertical position of patient to determine the maximal point of soft tissues ptosis of lower third of the face and submandibular area. Then tissue composition was determined using ultrasound without compression in this position. Parameters were measured: surplus subcutaneous and subplatysmal adipose tissue and its thickness, the thickness of platysma and the level of divergence of the edges of platysma from the mandible edges, ptosis platysma strands. Composition of tissue disturbing the contour of lower jaw from both sides was defined in the same position of patient. The thickness of the skin, subcutaneous fat and ptosis of muscles were assessed, i.e. the degree of participation of each tissue in age-related deformities.

## RESULTS

During our work we noted great diagnostic value of ultrasound examination of involutional changes of soft tissues of the lower third of the face and neck to determine all the causes of contour deformities and to define minimally invasive methods for their correction. Some clinical cases are present for illustration.

### Clinical case #1

**Fig.1:**
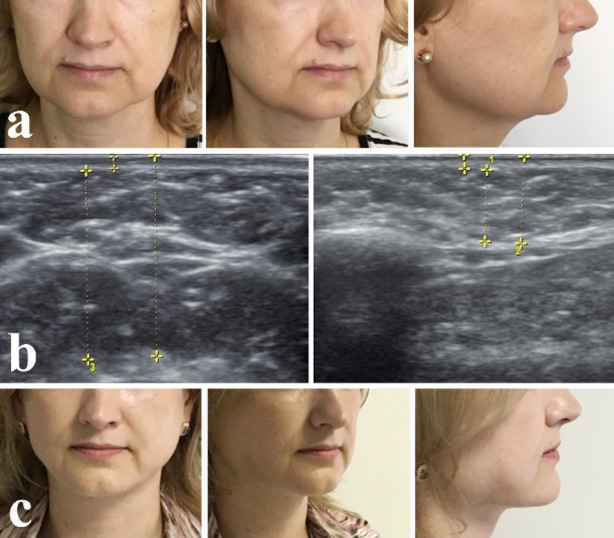
Patient before treatment (a) Accumulation of fat in the submandibular area, contour of the lower jaw (b), Patient month after surgery (c).

Patient H, 45 years old, complained of ptosis of soft tissue face and neck, violation of aesthetic. Clinical diagnosis: Age-related soft tissue atrophy of lower third of the face and neck. During examination we found slight sagging of soft tissues in the lower third of the face and submandibular area. There were slight excess skin and wrinkles with possibility of correction without excision and formation of deforming relief (Fig.1a).

On ultrasound examination accumulations of adipose tissue was found in the lower third of the face, neck and chin (73% of the thickness of soft tissue that violated contours of the lower jaw and the neck-submandibular angle). There were no atrophic age-related changes in platysma (Fig.1b).

Result of ultrasound examination showed that the local removal of excess subcutaneous fat in areas of disturbed contours of lower jaw and submandibular area was enough to correct the aesthetic lines of these areas, it was confirmed by the results of treatment of the patient (Fig.1c).

### Clinical case #2

**Fig.2:**
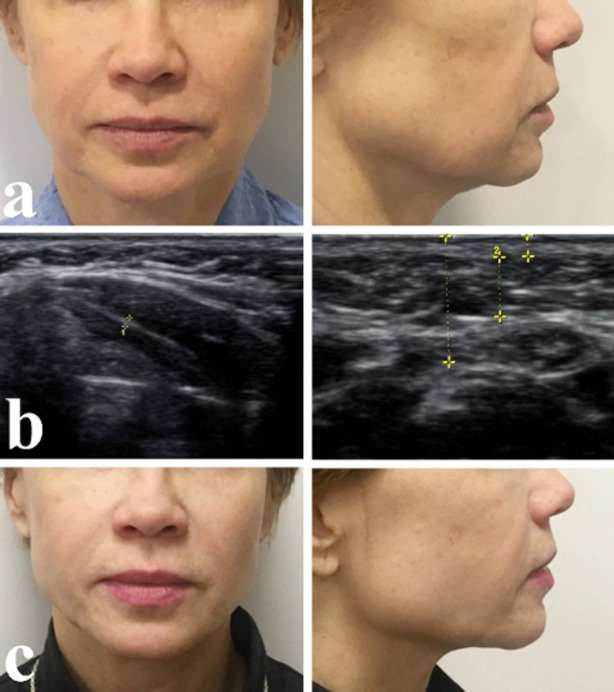
Patient before surgery (a), Strands of platysma, slight accumulation of fat tissue in the submandibular area (b), Patient 3 months after surgery (c).

Patient G., 54 years old, complained of soft tissue ptosis of the face and neck. Clinical diagnosis: Age-related soft tissue atrophy of lower third of the face and neck. During examination we found slight sagging of soft tissues in the submandibular area and platysma strands, painless and moderately displaced by palpation. There were excess skin and wrinkles with possibility of correction without excision and formation of deforming relief (Fig.2a).

On ultrasound examination in the vertical position of the patient we found ptosis of platysma strands from both sides, causing deformation of the contours of neck-submandibular angle, accumulation of fat tissue over platysma was inconsiderable and did not exceed the thickness of subcutaneous fat in adjacent regions. It was found that 68% of the thickness was determined by excessive deposition of subcutaneous fat.

Result of ultrasound examination showed that violation of contours of the neck-submandibular area was caused by age-related involution of tissues of subcutaneous neck muscle (platysma) and was not caused by local excessive accumulations of subcutaneous fat or skin ptosis. Violation of contours of lower jaw was mainly by local excessive accumulation of subcutaneous fat, with secondary unexpressed ptosis and excess skin (Fig.2b). Thus, closed platysmotomy was preferred with minimally invasive technique for correction of age-related deformation of neck and chin area, and contour liposuction was sufficient to correct these changes (Fig.2c).

### Clinical case #3

**Fig.3:**
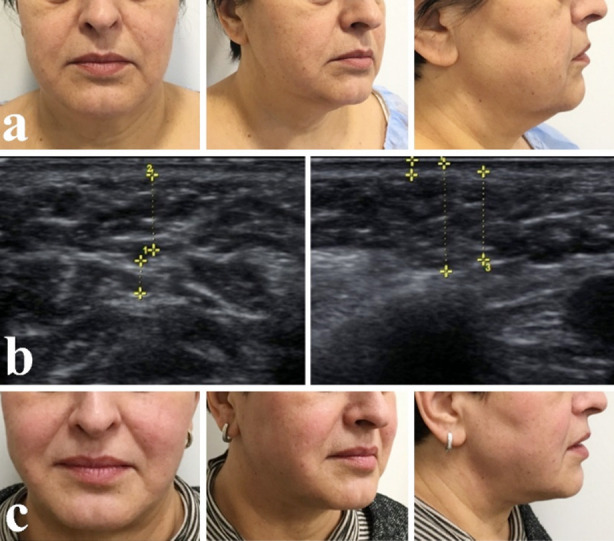
Patient before treatment (a) Accumulation of adipose tissue over the platysma, along the contour of the lower jaw, strands of platysma (b) Patient 3 months after operation (c).

Patient K., 58 years old, complained of ptosis of soft tissue face and neck, problems of aesthetic. Clinical diagnosis: Age-related soft tissue atrophy of lower third of the face and neck. Visually, there was a sagging of soft tissues in the submandibular and cervical area, excessive accumulation of adipose tissue, violation of the contour of the lower jaw, and a smoothed neck- submandibular angle (Fig.3a).

On ultrasound examination in the vertical position of the patient we found excessive accumulation of adipose tissue above the platysma in the chin and neck area problems of lower jaw contour due to accumulation of fat, ptosis of medial platysma was diagnosed. There were not determined either visually on palpation during physical examination (Fig.3b).

Based on the results of ultrasound, it was concluded that age-related problems were caused by local excess deposition of subcutaneous fat and ptosis of platysma cords. Thus, liposuction and platysmotomy was performed with intraoperative ultrasound control which were sufficient to correct age-related changes in this area for our patient (Fig.3c).

## DISCUSSION

Success of aesthetic surgery considerably depends on understanding of mechanisms of each specific deformity formation, skills to evaluate them in planning and making surgical correction as well as rehabilitation measures in postoperative period.^6^-^8^

Aging process affects all parts of the face and neck. Deformation in neck area can be determined by several factors: weakness of skin and platysma, appearance of platysma bands, ptosis of submandibular glands, lipodystrophy and even jaw resorption. Changing of face and neck proportions, quality and structure of skin, volume and location of fat make some interventions more difficult and may limit to obtain positive results.^9^-^11^

Analysis reveals that only few studies have been performed for objective diagnosis of involutional changes caused in lower third of face and neck. Unfortunately, practically all authors rely on subjective assessment of clinical situation. At the same time, based on anatomical studies, it is evident, that it is necessary to differentiate specific anatomical structures of soft tissue deformations. It is also difficult in many cases on general physical examination, especially in patients with thick skin and expressed subcutaneous fat cellular.

For example, it is not rare situation in such patients, when liposuction eliminate excess fat, but contours of neck-submental angle were deformed by platysma bands that became visual after surgery, on ptosis of all platysma.

Many authors in their works note the importance of accurate assessment of causes of neck contour disorders. Nevertheless, they often neglect to use diagnostic methods. Only lately publications in the world literature appeared about role of instrumental examinations in planning surgical correction of facial and neck tissue atrophy.

Japanese authors tried to use computed tomography for objective visualization and evaluation of age-related soft tissue changes of face and neck. Okuda I. et al. in their study (57 women aged 21-57 years), in which they used multi-detector row computed tomography (MDCT) for diagnostic.^12^ The superficial muscular aponeurotic system (SMAS) weakness index was measured using CT scans, then interconnection between SMAS weakness index and age of patient was analyzed. This research showed detailed facial changes associated with aging: weakness index and morphological changes in face were less in younger patients, and increase of age correlated with deeper morphological changes. Despite excellent visualization, CT is expensive, impossible to examine the patient in vertical position and control of actions during surgery.

Alfen N. V. was one of the first to use ultrasound to examine facial muscles in patients with myotonic dystrophy, and proved that this is perspective non-invasive method of diagnostics and monitoring in neuromuscular disorders. Ultrasound can be used for detecting early or subclinical face muscles lesion in Duchenne muscular dystrophy and Moebius syndrome to determine certain facial muscles are absent or atrophic.^13^

Volk G. F. et al. in their research described diagnostic value of ultrasound in lesions of facial nerve after injuries and tumors, that lead to atrophy of face muscles. Ultrasound was used to control regeneration and sizes of face muscles after reconstructive surgery, thickness and intensity of echo in patients with chronic peripheral facial paralysis at different stages of denervation and reinnervation. This method simultaneously presented functional and structural information about muscle state.^14^,^15^

Safran T et al. noted the importance of using ultrasound in cosmetology. They measured muscle thickness after botulism toxin injection, skin thickness after implantation of polylactic acid filaments and before eyelid and face surgeries. Their research indicated the importance of patients’ anatomy visualizing in real time for low complication rate (Global Aesthetic Improvement - approved scale of evaluating procedures for face) without radiation exposure.^16^

X. Wortsman, and colleagues have described in detail the ultrasound anatomy of face for non-invasive cosmetic and plastic surgery, pointed to the importance of ultrasound examination of facial structures in various deformities, paresis muscles and bruxism.^17^ However, examinations of patients were conducted in supine position, with absence of natural soft tissue ptosis.

G. Mashkevich et al. used ultrasound examination of the submandibular area in 10 patients. It helped to identify, which of this area components mainly caused deformation, especially in patients with so-called “heavy” necks.^18^

Analysis of works reveals that ultrasound is a non-invasive, not inexpensive method in clinical practice that can visualize in detail the anatomy of soft tissues of face. However, it was not previously used in clinical practice for diagnostic involute changes of face lower third and neck to determine treatment tactics.

New idea and finding of our work are the use of ultrasound examination for age-related changes of soft tissues of the lower third of face and neck in the vertical position of the patient. Different ptosis tissues and the degree of their participation in age-related changes are visible in different anatomical areas. It significantly help in determination of effective minimally invasive treatment methods in a number of clinical situations.

## CONCLUSIONS

Ultrasound examination is non-invasive, harmless, clinically available, inexpensive examination that allows to determine exact localization and degree of participation of different soft tissues of face and neck in age-related changes. It is very important for planning of use different minimally invasive techniques for facial and neck rejuvenation.

### Authors’ Contribution:

**VS:** Conceived, designed the study, data collection, editing of manuscript, review and final approval of manuscript.

**SA, AT, LS:** Did statistical analysis, data collection and manuscript writing.

## References

[1] Sahinoz T, Sahinoz S (2020). Investigation of healthy living strategies in elderly who achieved to live long and healthy. Pak J Med Sci.

[2] Giordano P, Mateu J, Rouif M, Laurent B (2011). Difficult necks. Diagnosis and treatment. Retrospective study of 145 cases using the method of Feldman. Anchir Plast Esthet.

[3] Tiryaki KT, Aksungur E, Grotting JC (2016). Micro-Shuttle Lifting of the Neck:A Percutaneous Loop Suspension Method Using a Novel Double-Ended Needle Aesth Surg J.

[4] Hegazy AM, Farouk A (2017). Simplified Method for Management of Platysmal Bands:Platysmotomy as an Office Procedure. Aesth Plast Surg.

[5] Balogh B, Frtihwald F, Millesi W, Millesi H, Firbas W (1988). Sonoanatomy of the muscles of facial expression. Surg Radiol Anat.

[6] Iankovan V (2016). Recent Advances in Face Lift to Achieve Facial Balance. J Maxillofac Oral Surg.

[7] Roy S, Buckingham E (2017). The Difficult Neck in Face lifting. Facial Plast Surg.

[8] Rouif M, Bogaert P (2017). Clinical analysis before surgery in facial and neck rejuvenation. Ann Chir Plast Esthet.

[9] Coleman SR, Grover R (2006). The Anatomy of the Aging Face:Volume Loss and Changes in 3-Dimensional Topography. Aesthetic Surg J.

[10] de Castro CC, Aboudib JH, Roxo AC (2012). Updating the concepts on neck lift and lower third of the face. Plast Reconstr Surg.

[11] Smith RM, Papel RM (2018). Difficult Necks and Unresolved Problems in Neck Rejuvenation.

[12] Okuda I, Yoshioka N, Shirakabe Y, Akita K (2019). Basic analysis of facial ageing:The relationship between the superficial musculoaponeurotic system and age. Exper Dermatol.

[13] Alfen NV, Gilhuis HJ, Keijzers JP, Pillen S, Van Dijk JP (2013). Quantitative facial muscle ultrasound:feasibility and reproducibility. Muscle Nerve.

[14] Volk GF, Wystub N, Pohlman M, Finkernsieper M, Chalmers HJ, Guntinas-Lichius O (2013). Quantitative ultrasonography of facial muscles. Muscle Nerve.

[15] Volk GF, Pohlman M, Sauer M, Finkernsieper M, Guntinas-Lichius O (2014). Quantitative ultrasonography of facial muscles in patients with chronic facial palsy. Muscle Nerve.

[16] Safran T, Gorsky K, Viezel-Mathieu A, Kanevsky J, Gilardino MS (2018). The role of ultrasound technology in plastic surgery. J Plast Reconstr Aesth Surg.

[17] Wortsman X, Ferreira-Wortsman C, Quezada N (2018). Facial Ultrasound Anatomy for Noninvasive Cosmetic and Plastic Surgery Procedures. Atlas of Dermatologic Ultrasound.

[18] Mashkevich G, Wang J, Rawnsley J, Keller GS (2009). The Utility of Ultrasound in the Evaluation of Submental Fullness in Aging Necks. Arch Facial Plast Surg.

